# The effect of Quinoa flour and enzymes on the quality of gluten‐free bread

**DOI:** 10.1002/fsn3.1527

**Published:** 2020-03-30

**Authors:** Saadat Azizi, Mohammad Hossein Azizi, Roxana Moogouei, Peyman Rajaei

**Affiliations:** ^1^ Department of Food Science and Technology Islamic Azad University North Tehran Branch Tehran Iran; ^2^ Department of Food Science and Technology Tarbiat Modares University Tehran Iran; ^3^ Department of Environmental Planning, Management and Education Islamic Azad University North Tehran Branch Tehran Iran; ^4^ Department of Food Science and Technology Islamic Azad University Varamin‐Pishva Branch Varamin Iran

**Keywords:** celiac, gluten‐free bread, lipase, protease, quinoa

## Abstract

Gluten‐free products usually are produced by refined flours such as rice and corn flour, which the bran is separated during processing. These flours are not nutritionally as rich as gluten containing products. Moreover, gluten‐free bread has several technical problems such as unfavorable texture, low volume, quick staling, and weaker color and taste compared with the wheat flour products. In this research, gluten‐free bread with various substitution of quinoa (0%, 15%, and 25%) was produced and the effects of lipase and protease enzymes on the quality of bread were investigated. The gluten‐free bread properties like physicochemical properties, rheological properties, and bread microstructure were evaluated. Moreover, the sensorial properties were assessed. The results have demonstrated that gluten‐free bread with quinoa flour has favorable properties. Also, lipase and protease enzymes could improve the quality of the bread containing quinoa. Protease and lipase enzymes increased the bread volume, specifically in sample containing 15% quinoa substitution. Moreover, the staling was delayed in sample 25% quinoa substitution. The bread was accepted by consumers, and the highest score belonged to 25% substitution of quinoa flour.

## Graphical abstract

All produced breads had suitable sensational and rheological properties. It is possible to produce promising gluten‐free bread by 15% and 25% quinoa flour. Lipase and protease enzymes could improve the quality of gluten‐free breads which are containing quinoa flour.

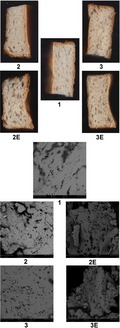

## 1 INTRODUCTION

Bread is one of the most crucial foods which is used daily in all the world. Wheat (*Triticum aestivum*) is a prominent cereal because of its gluten protein fraction. This protein is responsible for the unique viscoelastic properties of wheat dough and hence for the exceptional bread‐making potential (Hager et al., 2012).

Celiac disease is a common digestive disease, influences one percent of the population in the world. Prolamins in wheat, rye, and barley are major reasons for digestive problems in subjects with celiac disease. The treatment of this disease is only possible by avoiding gluten in daily diet. Gluten sensitivity represents the majority of food intolerance. Therefore, baking bread without gluten is crucial (Bourekoua et al., 2018; Turkut, Cakmak, Kumcuoglu, & Tavman, 2016). Today, about 15% of people in the world look for gluten‐free products, and their reason for a gluten‐free diet is not only because of celiac disease, but also for having a healthier diet. Among gluten‐free products, demand for bread is more than the others (Encina‐Zelada, Teixeira, Monteiro, Gonzales‐Barron, & Cadavez, 2018). Baking high‐quality bread requires gluten protein. Gluten protein makes the final structure of bread. Gluten maintains the gas bubbles in the bread and makes the favorable volume and texture for the bread. Eliminating the gluten from bread dough causes serious problems such as decreasing the cohesiveness, elasticity, and gas maintainability in the dough. Therefore, gluten‐free bread has less volume, poor flavor, friable texture, and high retrograding rate rather than wheat bread (Bourekoua et al., 2018). In celiac disease, gluten producing damages in the intestinal mucosa through inflammation of the microvilli, hence the ability of absorbing nutrients becomes deranged (Encina‐Zelada et al., 2018). On one hand, gluten‐free products are usually made by refined flour which often does not have nutrient content (Turkut et al.., 2016). On the other hand, subjects who have celiac disease should be encouraged to use rich and nutrient food. There are some alternatives for gluten containing cereals such as quinoa and Amaranth. These grains have high nutrition value, and using them in a gluten‐free product, increase the variety and quality of these products (Alvarez‐Jubete, Auty, Arendt, Gallagher, & Technology, 2010). Quinoa consumption has been recommended because of high‐quality protein, fibers, and minerals (such as iron and calcium). Quinoa flour has high sugar content (glucose and fructose which have a low glycemic index), dietary fibers, omega‐3 fatty acids, and phenolic compounds (especially flavonoids). Thus, quinoa flour could be used as a functional compound in gluten‐free bread (Turkut et al., 2016).

Most studies have investigated nutritional value, chemical composition, and baking quality of quinoa bread. However, the effect of additives such as lipase and protease enzymes on the quality of quinoa bread has not studied. Using enzymes in the baking industry could improve the physicochemical and rheological properties of dough and bread. Enzymes are naturally present or deliberately added to the food as processing aids. A wide range of enzymes exist, and their blend depends on the effect and application. Enzymes are used as technological aids in different stages of baking because they are effective in reducing the firmness of crumb, delaying the staling of baking, improving dough‐handling properties, and enhancing the bread quality. In addition, the enzymes are a better and safe alternative for chemical additives (Romano et al., 2018).

Lipase breaks down and degrades the ester bonds. Lipase is a good alternatives for emulsification in food products (Gerits, Pareyt, Decamps, Delcour, & Safety, 2014). Considering the beneficial effects of lipase in the production of bakery products, it seems that a higher quality of the product will be obtained after using these enzymes in gluten‐free products of quinoa.

Protease has ability to hydrolyze the peptide bonds in proteins. Protease could improve the quality of gluten‐free bread. Protease improves the expansion ability of dough starch and gives cohesiveness for bread texture during baking process. Therefore, changes in the gluten‐free dough profiles can be related to the modifications in protein–starch interactions resulted from the proteolytic activity.

In such conditions, protein structures surrounding the starch granules give rigidity to the paste, and the rheology of the system is created by the rigidity of the suspended particles (Renzetti & Rosell, 2016). Protease reduces starch interactions by decomposing starch granules and gives suitable porosity, higher volume, and softer crumb to the bread. Protease reduces bread staleness by decreasing the interaction of starch–starch between starch granules. Treatment of gluten‐free bread by protease increased the stability of many microbubbles during fermentation in comparison with the control sample (Hamada, Suzuki, Aoki, & Suzuki, 2013; Kawamura‐Konishi, Shoda, Koga, & Honda, 2013; Renzetti & Arendt, 2009). In this research, gluten‐free bread with 0%, 15%, and 25% quinoa substitution was produced, and the effects of lipase and protease enzymes as a quality improver were also evaluated. In the present study, bread physicochemical and rheological properties, color and porosity, dough yield and bread yield percentage, microstructure, and sensory properties were investigated.

## 2 MATERIAL AND METHODS

### 2.1 Chemical and reagent

Quinoa was cultivated in Peru and purchased from local market in Tehran, Iran. Some common materials like rice flour, sugar, and oil were prepared from the local markets in Tehran, Iran. Corn starch was donated by Glokozan. Sodium caseinate was purchased from Pegah, inulin was purchased from Sensus, transglutaminase enzyme was purchased from Siveele, protease and lipase enzymes were purchased from Novozymes, DATEM was purchased from Pars Behbood, xanthan gum was purchased from Gum Tech, baking powder was purchased from Golha, and active dry yeast as a vacuumed package was purchased from Razavi Yeast Company.

### 2.2 Bread making

Components of each formulation were prepared based on Table 1. The critical basic formulation was determined empirically by trial and error. Quinoa seeds were washed by cold water until yellow color and foam were removed (saponin is responsible for the bitter taste of quinoa). Then, the samples were dried to 10% moisture, and the dried seeds were milled using a laboratory blender until the flour could pass through a 0.5 mm stainless steel sieve. In this study, 1 u/g pro transglutaminase enzyme based on the basic protein of each treatment was calculated and used. In each formulation, 1.35 g xanthan gum was utilized. The optimal rate of this gum was detected by trial and error. All used ingredients and the rates in the five formulations are presented in Table 1. For bread, powder materials of each formulation (except xanthan gum, sugar, and yeast) were mixed. The water for making the dough was about 87% of materials (based on powder content in each formulation) for all formulations. Therefore, about 40 ml of water was used for activating yeast with sugar. The remained water was added to powder materials and mixed slowly for 10 min. After primary mixing, oil in each formulation was added to the dough and mixed for 1 min. Then, xanthan gum was added to the dough and mixed by a fast speed of blender for 5 min. The prepared dough was equally distributed in small tins of 10 × 5×3 cm. Then, dough tins were incubated at 35°c temperature and 85% humidity for 40 min. After incubation, the samples were baked in an electric oven at 170°C temperature for 30 min. To protect the bread from drying and keeping humidity in the oven atmosphere, a pan of water was placed into the oven. After baking, the bread samples were cooled down at room temperature for an hour. After cooling, to prevent moisture reduction, the samples were kept in the polyethylene package at 25°C until the experiment. Except for staling, all other measurements were indicated on the day of sample baking, and for measuring staling, the samples were stored at room temperature for 72 hr.

**Table 1:** Formulation ingredients (g)

Ingredients	1	2	3	2E	3E
Rice flour	55	46.75	41.25	46.75	41.25
Corn starch	35	35	35	35	35
Sodium Caseinate	6	6	6	6	6
Inulin	3	3	3	3	3
Sodium chloride	2	2	2	2	2
DATEM	0.07	0.07	0.07	0.07	0.07
Xanthan gum	1.35	1.35	1.35	1.35	1.35
Baking powder	2	2	2	2	2
Sugar	10	10	10	10	10
Active Dry Yeast	2	2	2	2	2
Sunflower oil	10	10	10	10	10
Transglutaminase enzyme	4	4.26	4.75	4.26	4.75
Quinoa flour	0	8.25 (15%)	13.75 (25%)	8.25 (15%)	13.75 (25%)
Protease enzyme	0	0	0	0.01	0.01
Lipase enzyme	0	0	0	0.01	0.01

**Figure 1:**
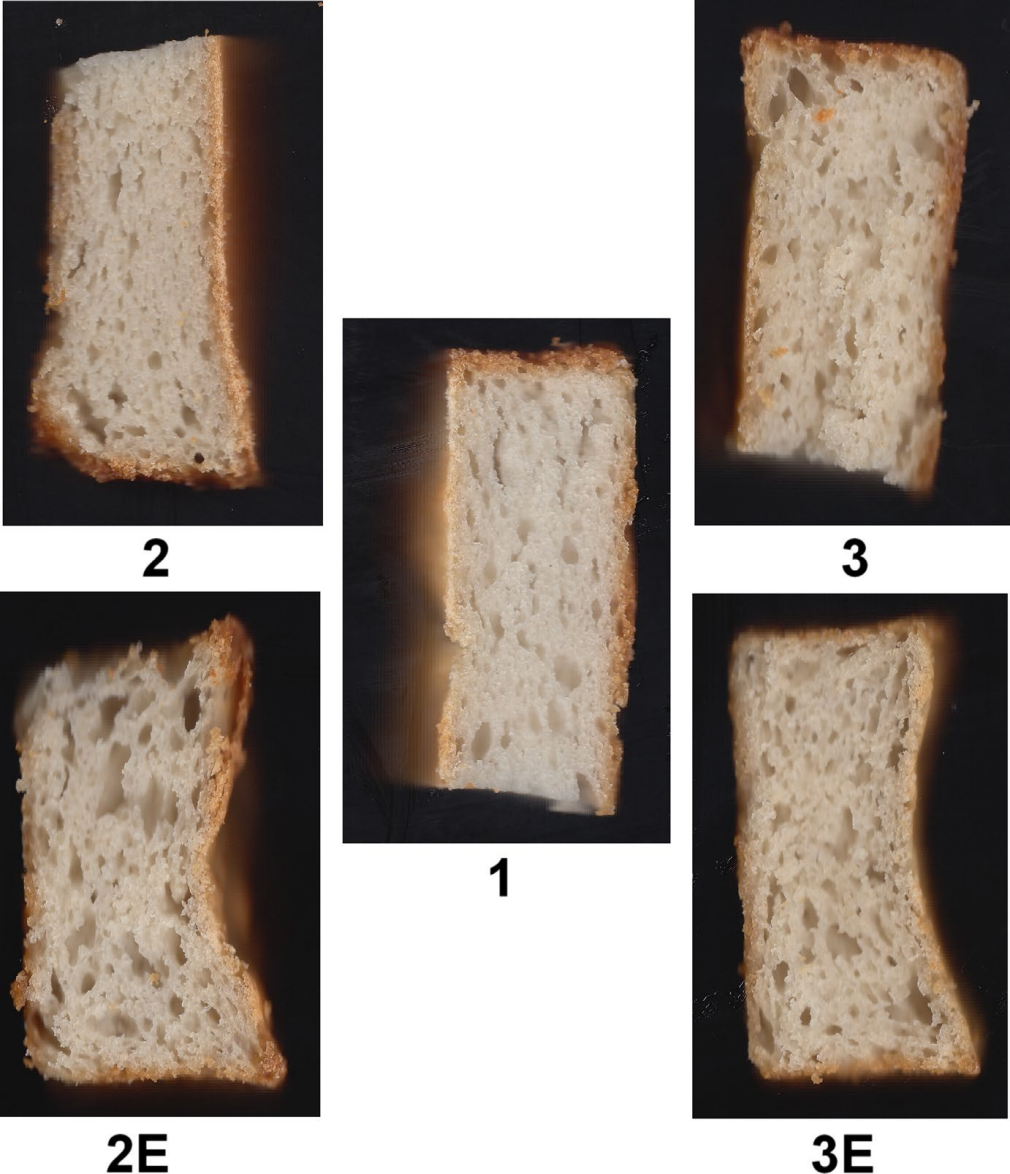
Bread crumb image of each formulation

Samples were divided into five groups. The sample (a) considered as the control, and the samples (b) and (c) were 15% and 25% quinoa substitution, respectively. The samples (2E) and (3E) were 15% and 25% quinoa substitution, respectively, but both containing protease and lipase enzymes in their formulation (Figure 1).

### 2.3 Physicochemical Analysis of the bread

Moisture content was measured based on AACC methods (01‐44) and by AND moisture detector (X‐50 model) (AACC, 2000). Ash was measured based on AACC approval methods (08‐01) (AACC, 2000). Bread volume was measured by the movement of millet seeds and based on AACC methods (10‐05) (AACC, 2000). Bread specific volume determined by dividing bread volume (ml) to its mass (g) and based on (ml/g) (Kawamura‐Konishi et al., 2013).

The color of bread crumb was measured by digital image analysis method. The analysis was applied after 2 hr of baking time. In this method, the luminance index (L*), red or green index (a*), and blue and yellow index (b*) were indicated. The L* index illustrates the luminance or lightness component of the sample and changes from zero (absolute darkness) to a hundred (absolute whiteness). The a* indicates the content of red or green with a range of −120 (pure green) to +120 (pure red). The b* defines the content of yellow and blue, and its rate is from −120 (pure blue) to +120 (pure yellow). For measuring the indicators, two slices of bread (2*2 centimeters) were cut from a crumb of bread. Then, a picture (with the resolution of 300 pixels per in^2^) was taken by the scanner (Cano Scan LIDE 110), and the L*, a, and b* were calculated by *Image J* software layout and by selecting *LAB* section in *Plugins* tab (Zheng, Sun, & Zheng, 2006).

Bread porosity was determined by the image analysis technique. The image analysis method was applied after 2 hr since baking. First, a 2 × 2 centimeters slice was prepared from bread crumb. Then, a picture (with the resolution of 300 pixels per in^2^) was taken by the scanner (Cano Scan LIDE 110). All images were analyzed by Image J software. Gray images were taken by activating an 8‐bit image. In order to produce a gray image to a binary image, the binary part of the software was activated. These images consist of bright and dark points in which the rate of bright points to dark points indicates the porosity of bread. The high rate of this ratio illustrates more holes and cavity in bread. The ratio was calculated by activating the analysis tab in the software, and the porosity of the samples was indicated (Haralick, Shanmugam, & Dinstein, 1973).

Dough yield (%) was measured by the amount of obtained dough (g) from 100 (g) of raw materials (Mohammadi, Azizi, Neyestani, Hosseini, & Mortazavian, 2015).

The bread yield (%) was evaluated according to the following equation (Mohammadi et al., 2015):

(1) $$\text{Bread yield percentage:}\;\left(\text{Bread mass/Flour mixed mass}\right)\;\times 100$$

Rheological properties such as hardness, springiness, chewiness, and cohesive were analyzed by using a Zwick/Roell texture analyzer (BT1_FR0.5TH.D14). The curves were illustrated by the force–time curve device. Three‐cycle test was defined and used for this analysis. Compression time and rest time in each cycle were determined as 60 and 20 s, respectively. The analyzer load cell capacity was 500 N, and the diameter of the aluminum probe used for analyses was 10 mm. The aluminum probe progressive speed was 2.5 mm/s (150 mm/min), and the primary load cell was 0.5 N.

Many studies have indicated that gluten‐free products have more staling rate than wheat flour products. Quick staling is common in starch‐based bread. When there is no starch–protein bonding, the interaction of starch polymers becomes quicker and, consequently, accelerates the crystallization and retrogradation of starch polymer. In this situation, water bonding is weak and water transferring from crumb to crust will be accelerated which reduces bread quality during shelf life (Encina‐Zelada et al., 2018; Hager et al., 2012; Korus, Witczak, Ziobro, Juszczak, & Technology, 2015).

In this research, the firmness measured and determined in 24 hr and 72 hr after backing. Bread staling rate was calculated according to the equation below:

(2) $$\text{Bread staling rate:}\left(\frac{\text{crumb hardness 72 hour after baking-crumb hardness 24 hour after baking}}{\text{crumb hardness 24 hour after baking}}\right)$$

### 2.4 Microstructure

Scanning electron microscopy (SEM) was selected to investigatethe microstructure of bread. Slices were cut from bread. Then samples were freeze‐dried for approximately 24 hr in the laboratory freeze dryer. In the next step, samples were shortly grinded with mortar and pestle and then attached onto double‐sided carbon tape and fixed to an aluminum specimen stub and were preliminary gold‐coated. Then, the electron images were acquired at an accelerating voltage of 10 kV by SEM device (Prox model and from Phenom Company). X2000 magnification images were taken from bread crumb.

### 2.5 Sensorial properties of the bread

Sensorial properties of the bread were examined by 5 trained assessor and by numeral scoring. First, two bread slices of each formulation coded with three‐digit numbers were given to the assessors to score each formulation from 1 to 5, individually and in separate rooms. Separated rooms had normal daylight. A bottle of water was given to the assessors to drink before testing each new sample. The bread texture softness, elasticity, chewing ability, color, porosity, taste, smell, and the overall score was evaluated twice by each assessor (Mohammadi et al., 2015).

### 2.6 Statistical analysis

The obtained results of the current study were analyzed in SPSS v.22 (SPSS Inc.). All experiments were performed in three replicates. ANOVA was used for data analysis, and Duncan's multiple range test was used to compare the means. The graphs were drawn using Excel 2013 software. The p value of 5% was considered as significant.

## 3 RESULTS AND DISCUSSION

### 3.1 Physicochemical analysis of the bread

The control sample had the lowest moisture content. All (2), (3), (2E), and (3E) bread samples had significantly more moisture than the control sample. This probably related to the increase in fiber content and thence moisture content in bread samples which was because of quinoa in the bread (Table 2).

**Table 2:** The quality parameters of the gluten‐free bread

Dough yield	Bread yield	Specific volume	Volume	Ash	Moisture	Treatment
171.0 ± 0.76^a^	131.0 ± 1.35^b,c^	2.77 ± 0.06^c^	17.8 ± 0.02^a^	3.1 ± 0.02^a^	31.0 ± 0.05^a^	**1**
178.4 ± 7.66^a^	128.2 ± 1.42^a,b^	2.11 ± 0.01^a^	18.9 ± 0.01^b^	3.12 ± 0.03^b^	32.0 ± 0.03^b^	**2**
168.0 ± 0.76^a^	132.2 ± 0.03^c,d^	2.39 ± 0.02^b^	33.3 ± 0.04^d^	3.124 ± 0.04^d^	32.7 ± 0.02^b^	**3**
165.3 ± 0.02^a^	125.9 ± 0.05^a^	2.89 ± 0.03^d^	40.2 ± 0.01^e^	3.121 ± 0.01^c^	31.8 ± 0.4^b^	**2E**
168.0 ± 0.03^a^	134.4 ± 0.02^d^	2.39 ± 0.01^b^	31.8 ± 0.03^c^	3.125 ± 0.02^e^	32.4 ± 0.05^b^	**3E**

Note

The difference in numbers with the same letters is not statistically significant on the basis of Duncan's test.

Ash content in all samples was not significantly different (Table 2). Bread volume is one of the most qualitative parameters, which affects the costumer's choices. The gas bubbles exiting from bread texture during baking reduce the bread volume (Encina‐Zelada et al., 2018). Utilizing quinoa in formulation significantly increased the volume. Using 25% quinoa (sample 3) significantly increased the bread volume compared with sample (2) with 15% quinoa. The volume of the sample (2E) was higher than the sample (2). Lipase and protease enzymes increased the volume of bread containing 15% quinoa, which is because of high gas retaining ability in bread texture. The volume of the sample (3E) was lower than the sample (3). Lipase and protease enzymes did not affect the volume of bread containing 25% quinoa. By increasing the amount of quinoa, the dough protein content, the enzyme's protein–starch bond, and texture hardness were increased. Consequently, hardness prevented the increase in bread volume. Lorenz and Coulter (1991) also reported that replacing 5% of the bread flour with quinoa flour increases the bread volume. They indicated that this increase was as a result of high alpha‐amylase activity in the quinoa flour. High activity of alpha‐amylase enzyme results in more fermentable sugars and thus increases the loaf volume. They claimed that bread samples with 5% and 10% substitution of quinoa had both inner and outer acceptable properties. The quinoa flour more than 10% decreased the loaf volume (Lorenz & Coulter, 1991). Morita, Hirata, Park, and Mitsunaga (2001) reported that 7.5%–10% quinoa flour substitute for wheat flour increased the volume of bread compared with the control sample. They also stated that adding quinoa flour to wheat flour improves the balance of starch, protein, and lipid while adding more than 15% quinoa flour substitute decreases the volume (Morita et al., 2001).

Specific volume is one of the primary qualitative parameters of bread. Specific volume is an important parameter mainly in costumer choices. The higher ratio of volume to the mass is a favorable property for the bread makers (Encina‐Zelada et al., 2018; Turkut et al., 2016). The specific volume in this research changed from 2.11 to 2.89 ml/g. In some studies, the specific volume of bread was reported about 4–5 ml/g which depends on the type of formulation, baking method, and using or not using sourdough. The specific volume of gluten‐free bread was reported between 1.33 and 2.4 ml/g in most studies. The specific volume of gluten‐free bread of rice, buckwheat, and 2% xanthan gum was reported about 1.9 ml/g (Encina‐Zelada et al., 2018).

The specific volume of sample (2) was remarkably less than the sample (3). As the amount of quinoa in the formulation increased, the specific volume increased, probably due to the increased monosaccharide content and increased yeast gas production.

The use of lipase and protease enzymes in the formulation containing 15% quinoa increased the specific volume of the bread, which is related to the stability of the gas bubbles in the dough texture. However, using enzymes in the formulation containing 25% quinoa did not affect the specific volume of the bread.

Many studies reported the negative effect of quinoa on the specific volume of bread (Bilgiçli & İbanoğlu, 2015; Iglesias‐Puig, Monedero, Haros, & Technology, 2015; Stikic et al., 2012). However, utilizing some hydrocolloids keeps the gas bubbles inside the bread matrix during fermentation. Therefore, the specific volume becomes the same as the control sample (Turkut et al., 2016).

In this research, by increasing quinoa in the formulation, the specific volume was significantly increased. This was in agreement with Föste et al. (2014) study. In Turkut et al. (2016) study, the rate of quinoa in formulation did not have significant effect on the specific volume of gluten‐free bread (Turkut et al., 2016).

Dough yield in all samples was no significantly different compared with the control sample (Table 2).

The bread yield of the samples (2) and (3) was not significantly different from the control sample. However, bread yield in the sample (3) was significantly higher than the sample (2). The bread yield of the sample (2E) was remarkably less than the control sample, while there was no significant difference between the sample (2E) and sample (2).

The bread yield of the sample (3E) was significantly more than the control sample. The bread yield of the sample (3E) was also significantly higher than the sample (2E). The results showed that enzymes did not affect the bread yield. However, 25% of quinoa increased the bread yield. This probably was because of the fiber content of quinoa, increased moisture‐binding capacity, and moisture maintains in the bread texture.

The color of bread is a result of complex chemical reactions between proteins and carbohydrates during the baking process. Moreover, the bread formulation itself could change the final bread color (Turkut et al., 2016). Digital image analysis of bread crumb indicated a visual difference between the formulations (Table 3).

**Table 3:** Image analyses parameters of the bread samples

Treatment	Crumb L*	Crumb a*	Crumb b*	Porosity (%)
1	74.25 ± 0.43^b^	2.38 ± 0.10^a^	10.38 ± 0.26^a^	21.20 ± 0.41^b,c^
2	69.40 ± 0.19^a^	3.43 ± 0.07^b,c^	12.90 ± 0.11^b^	19.90 ± 0.29^b^
3	71.13 ± 1.36^a^	3.89 ± 0.29^d^	15.37 ± 0.72^c^	22.93 ± 1.43^c^
2E	68.69 ± 1.86^a^	3.26 ± 0.23^b^	12.83 ± 0.93^b^	27.07 ± 0.85^d^
3E	69.68 ± 0.75^a^	3.81 ± 0.18^c,d^	15.61 ± 0.39^c^	16.13 ± 0.68^a^

Note

The difference in numbers with the same letters is not statistically significant on the basis of Duncan's test.

The range of L*is from 68.69 to 74.25. The control sample had the maximum amount of L*, and the luminance was significantly higher than the other samples (There was no significant difference between the other samples). It shows that applied quinoa in the formulation has reduced the L*. However, the amount of quinoa (15% or 25%) did not have a significant effect on L*. Moreover, using protease and lipase enzymes in formulation did not significantly change the L*. Using quinoa in formulation significantly reduced the luminance index (L*) which was because of applying less rice flour.

Gluten‐free bread is usually identified by lighter color because of starches and rice flour; thus, darker color in this bread is more favorable. Although some studies indicate that increasing the amount of quinoa flour statistically decreased the lightness values of crumb and crust (Alvarez‐Jubete et al., 2010; Bilgiçli, İbanoğlu, & o. F. S., & Technology., 2015; Lorenz & Coulter, 1991), but Turkut et al. (2016) did not observe such difference (Turkut et al., 2016).

The range of a* is from 2.38 to 3.89. The maximum index was observed in the sample (3), and the minimum rate was observed in the control sample. Adding 15% to 25% quinoa in the formulation was significantly increased a* and caused more red color in the bread. The enzymes did not have a significant effect on the a*.

The range of b* varied from 10.38 to 15.61. The maximum rate observed in sample (3E), and the minimum rate is related to the control sample. Increasing the quinoa content from 15% to 25% was significantly increased the b*. However, adding enzymes did not have a significant effect. The use of quinoa was increased a* and b* indicators in bread, probably due to the quinoa pigments.

Utilizing quinoa significantly changed the color of bread. Higher substitution of quinoa in formulation increased the yellow index which is due to the yellow color of the quinoa (Bilgiçli et al., 2015; Turkut et al., 2016). In Bilgiçli et al. (2015) study, a* and b* indexes were increased by adding quinoa to the formulation (Bilgiçli et al., 2015).

Bread porosity percentage changed from 16.13 to 27.07. The highest porosity belonged to the sample (2E), and the lowest porosity was in the sample (3E) (Table 3).

Using 15% quinoa in formulation did not change the porosity percentage. Porosity in the sample (3) was higher than the sample (2). This means that increasing the quinoa from 15% to 25% in the formulation has significantly increased the porosity. Adding protease and lipase enzymes to the sample (2) significantly increased the porosity. The porosity in the sample (3E) was significantly lower than the sample (3). Using protease and lipase enzymes in 25% quinoa content bread significantly lowered the porosity. By increasing the quinoa substitution and utilizing enzymes in the formulation, dough hardness was increased. Consequently, it prevented bread volume extension and porosity.

Bread springiness is related to its freshness and elasticity. The elasticity decreases following the decrease in bread springiness or resilience. Both properties show the ability to return the first shape after removing the stress (Cornejo, Rosell, & Technology, 2015). The range of springiness is between 0.47 and 0.59. Sample (2E) had the lowest springiness, and the highest springiness is related to sample (3). Utilizing quinoa and enzymes did not significantly change the bread springiness (Table 4).

**Table 4:** Rheology characterization of the bread samples

Treatment	Springiness	Chewiness	Cohesiveness	Hardness 24 hr	Hardness 72 hr	Staling
1	0.53 ± 0.02^a,b^	11.10 ± 1.55^b^	0.21 ± 0.04^b^	97.0 ± 1.4^e^	120.8 ± 2.1^e^	0.24 ± 0.02^c^
2	0.55 ± 0.01^a,b^	1.48 ± 0.09^a^	0.14 ± 0.01^a^	25.8 ± 0.5^a^	29.7 ± 0.8^a^	0.15 ± 0.03^a^
3	0.59 ± 0.04^b^	3.12 ± 0.24^a^	0.18 ± 0.03^a,b^	39.7 ± 3.64^b^	44.94 ± 2.1^b^	0.20 ± 0.04^b^
2E	0.47 ± 0.06^a^	3.62 ± 0.52^a^	0.14 ± 0.01^a^	56.2 ± 1.9^c^	64.6 ± 1.4^c^	0.15 ± 0.01^a^
3E	0.51 ± 0.03^a,b^	8.83 ± 0.42^b^	0.15 ± 0.02^a,b^	83.4 ± 1.1^d^	94.24 ± 1.3^d^	0.13 ± 0.03^a^

Note

The difference in numbers with the same letters is not statistically significant on the basis of Duncan's test.

The range of bread chewiness was between 1.48 and 11.10. The lowest chewiness was in the sample (2E), and the highest chewiness was in the control sample. The enzymes changed the chewiness only in the 25% quinoa content sample. The enzymes were not effective on the 15% quinoa content sample. This probably was because of more starch–protein or starch–lipid bonds (Table 4).

Cohesiveness characterizes the extent to which a material can be deformed before it ruptures and reflects the internal cohesion of the material. Bread crumb with high cohesiveness is desirable because it forms a bolus, instead of disintegrating during mastication, whereas low cohesiveness indicates bread will get ruptured easily (Encina‐Zelada et al., 2018).

The range of cohesiveness was between 0.14 and 0.21. The highest amount of cohesiveness was in the control sample, and the lowest amount was recorded for samples (2) and (2E). The cohesiveness of (2) and (2E) samples was significantly lower than the control sample. There was no significant difference between other samples and the control sample (Table 4).

The required force to compress foods between teeth is called hardness. The rate of the hardness is between 25.8 and 97. The maximum and minimum hardness were related to the control sample and the sample (2), respectively. The hardness of all samples was significantly lower than the control sample. The hardness of the sample (3) was significantly stronger than the sample (2). Hence with increasing the amount of quinoa from 15% to 25% in bread, the hardness was significantly increased. In the present study, increasing quinoa substitution from 15% to 25% has increased the hardness. The same result was observed in Turkut et al. (2016) study (Turkut et al., 2016). Using protease and lipase enzymes in 15% and 25% quinoa content bread significantly increased the hardness. The hardness of the sample (3E) was significantly more than the sample (2E). It can be concluded that in the presence of lipase and protease enzymes, by increasing the amount of quinoa substitution from 15% to 25%, the amount of starch–protein bond and consequently the dough and bread hardness increased.

The maximum rate and minimum rate of staling are related to the control sample and the sample (2E), respectively. The increase in quinoa substitution from 15% to 25% significantly has increased the staling. Lipase and protease enzymes only reduced the 25% quinoa substitution which is because of the decrease of the interaction of starch–starch and forming a new starch–protein compound. This result is similar to Kawamura‐Konishi et al. (2013) study. They claimed that the reason for staling reduction in bread related to using the protease in bread formulation, which increases the interaction of starch–protein instead of starch–starch interaction (Kawamura‐Konishi et al., 2013).

### 3.2 Microstructure

**Figure 2:**
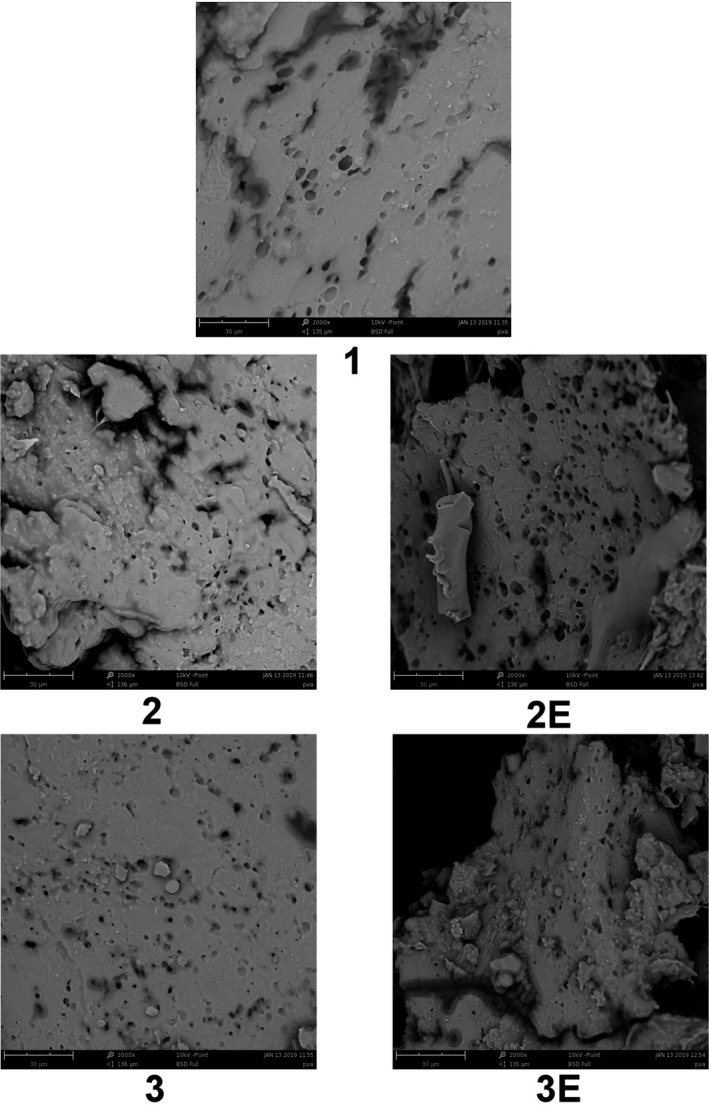
Scanning electron microscopy images of samples

Based on images of the control sample (1), starch granules were obvious and did not gelatinize. Texture cohesiveness was suitable, but porosity was low. In sample (2) images, starch granules were rather visible, but most of them were disintegrate. Texture cohesiveness was suitable (Figure 2).

In sample (3) images, starch granules were rather visible, but most of them were decomposed and gelatinized. Texture cohesiveness was better than the sample (2), and the texture was more homogenous. Texture porosity was favorable, and the gas cells were distributed adequately.

In the sample (2E) images, the polymeric fibers of protein were decomposed and a compound was made between starch granules and decomposed protein filaments. The texture had more porosity than other samples which was coincided with Digital Image Analysis and sensory analysis results. The texture was less cohesive compared with the control sample (1) and sample (2E), and also, it was more brittle. In sample (3E) images, a compound was made between starch granules and decomposed protein filament. The texture was less cohesive than the control sample (1) and sample (3).

### 3.3 Sensory evaluation of the bread

**Figure 3:**
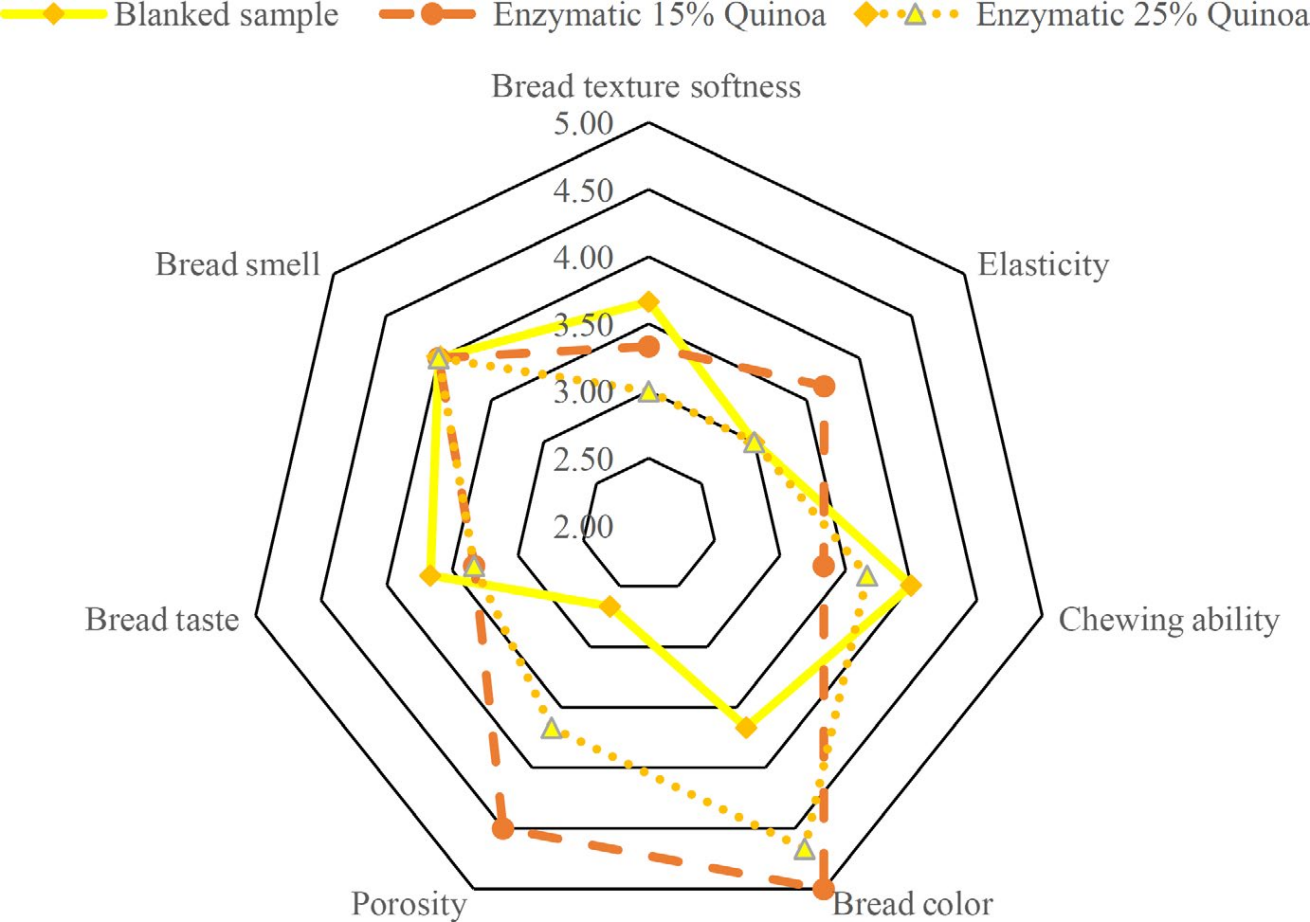
The sensory analysis of the bread contained different amount of quinoa

**Figure 4:**
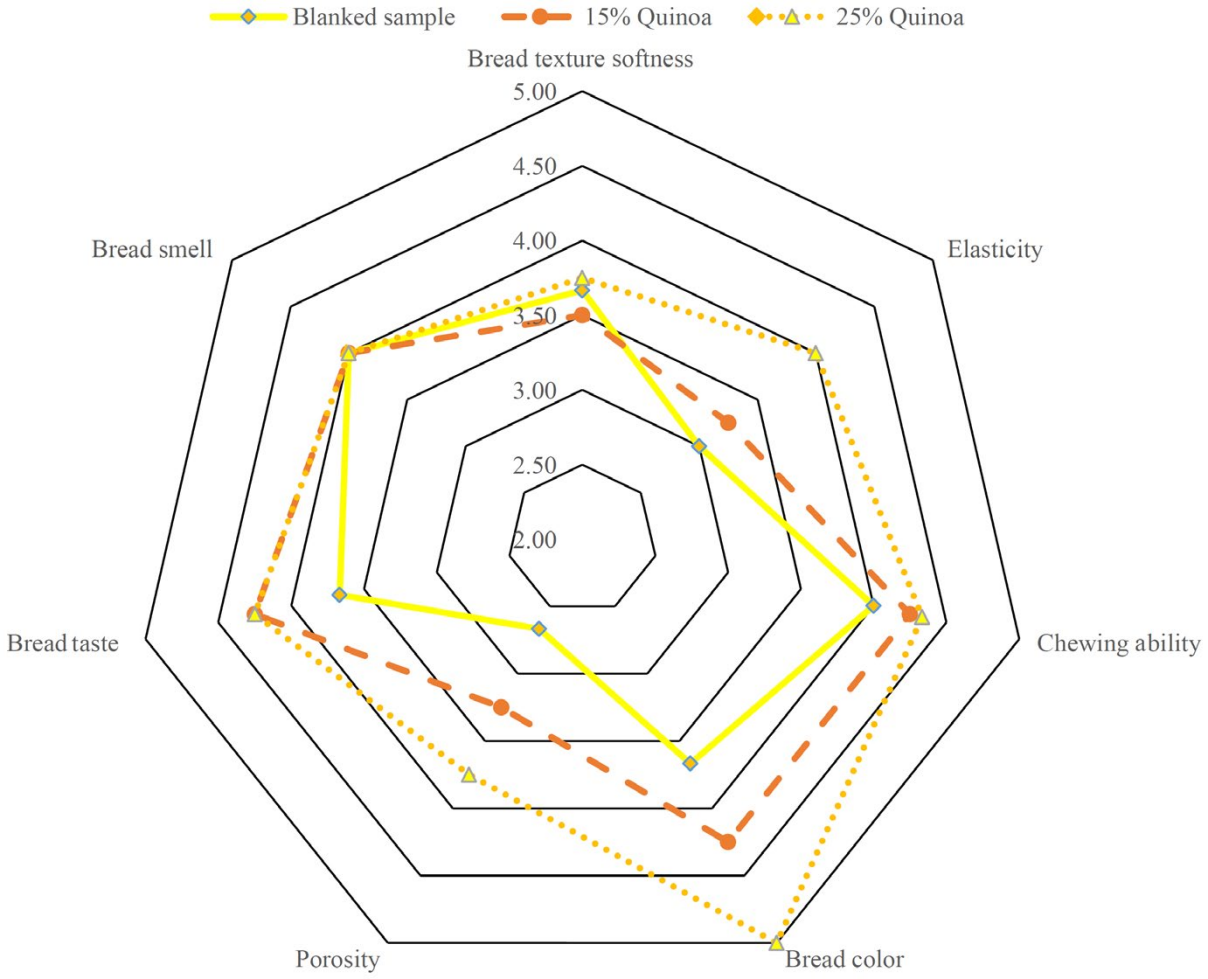
The sensory evaluation of the bread contained enzymes and different amount of quinoa

Sensory analysis is a crucial factor for an acceptable evaluation of applying quinoa and enzyme in the formulation. The sensory analysis result (Table 5) demonstrated that texture softness, elasticity, smell, and taste of all samples were not significantly different from each other. The samples (3) and (2E) had the highest score in the color test. This shows that using quinoa in the formulation has improved gluten‐free bread. Samples with substitution of quinoa had a darker crust color than the control sample. Gluten‐free bread is made based on rice flour and has a light yellow color which is not favorable. By applying the quinoa in the formulation of bread, the color could be improved. This result coincides with the Föste et al. (2014) study (Föste et al., 2014). In sensory analysis, the control sample had the lowest porosity and the sample (2E) had the highest porosity. This result is the same as the digital image analysis result. The samples (2) and (3) had the best taste (Figures 3 and 4).

**Table 5:** Results of the bread Sensory Analysis

Treatment	Bread texture softness	Elasticity	Chewing ability	Bread color	Porosity	Bread taste	Bread smell	Overall score
1	3.7 ± 0.47^a^	3.0 ± 0.82^a^	4.0 ± 0.82^a^	3.7 ± 0.47^a^	2.7 ± 0.47^a^	3.7 ± 0.47^a^	4.0 ± 0.82^a^	3.8 ± 0.47^a^
2	3.5 ± 0.5^a^	3.3 ± 0.43^a^	4.3 ± 0.83^a^	4.3 ± 0.83^a,b^	3.3 ± 0.83^a,b^	4.3 ± 0.43^a^	4.0 ± 0.71^a^	3.8 ± 0.83^a^
3	3.8 ± 0.43^a^	4.0 ± 0.82^a^	4.3 ± 0.47^a^	5.0 ± 0.1^b^	3.8 ± 0.43^a,b^	4.3 ± 0.43^a^	4.0 ± 0.1^a^	4.3 ± 0.43^a^
2E	3.3 ± 0.47^a^	3.7 ± 0.47^a^	3.3 ± 0.47^a^	5.0 ± 0.1^b^	4.5 ± 0.5^b^	3.3 ± 0.94^a^	4.0 ± 0.1^a^	3.7 ± 1.25^a^
3E	3.0 ± 0.1^a^	3.0 ± 0.82^a^	3.7 ± 0.47^a^	4.7 ± 0.47^a,b^	3.7 ± 0.47^a,b^	3.3 ± 0.47^a^	4.0 ± 0.82^a^	3.7 ± 0.47^a^

Note

The difference in numbers with the same letters is not statistically significant on the basis of Duncan's test.

All samples were acceptable for the consumer, and the most score belonged to sample (3). However, it was not notably higher than other samples. In Chlopicka, Pasko, Gorinstein, Jedryas, and Zagrodzki (2012) study, about 85% of consumers claimed that quinoa bread does not have an undesirable taste (Chlopicka et al., 2012). In Stikic et al. (2012) study, about 20% of consumers gave excellent scores. In this study, the researcher claimed that quinoa could be utilized for the fortification of many baking products (Stikic et al., 2012). In the Elgeti et al. (2014) study, using quinoa flour in formulation improved the color of gluten‐free bread. The bread with 30% of quinoa substitution did not have a suitable taste (Elgeti et al., 2014).

## 4 CONCLUSION

The results showed that producing a promising gluten‐free bread by quinoa is possible. Quinoa has more nutritional value than other refined cereals. All produced bread samples had suitable sensational and rheological properties. Moreover, enzymes could be applied as an improver of gluten‐free bread. Protease and lipase enzymes have increased bread volume and specific volume in 15% quinoa substitution bread. Consequently, applying enzymes for improving the quality of gluten‐free dough is a promising technology for gluten‐free baking products. Future studies should apply several pseudo‐cereals in various formulations for gluten‐free bread and investigate the rheological properties of their dough.

## CONFLICT OF INTEREST

The authors declare that they do not have any conflict of interest.

## ETHICAL STATEMENTS

This study does not involve any human or animal testing.
